# Nest Box Condition and Maintenance of Barn Owls (*Tyto alba*) in Tropical Oil Palm Plantations

**DOI:** 10.3390/ani16060881

**Published:** 2026-03-12

**Authors:** Sukanya Thongratsakul, Marnoch Yindee, Kriangsak Hamarit, Nirawat Sinnarong, Wallaya Manatchaiworakul, Worawidh Wajjwalku, Chaithep Poolkhet

**Affiliations:** 1 Akkhraratchakumari Veterinary College, Walailak University, Nakhon Si Thammarat 80160, Thailand; sukanya.tho@wu.ac.th (S.T.); marnoch.yi@wu.ac.th (M.Y.); 2 STS Green Co., Ltd., 3/23 Moo 5, Lat Sawai, Lam Luk Ka, Pathum Thani 12150, Thailand; hamarit101@yahoo.com; 3 Chumporn Palm Oil Industry Public Company Limited, 296 Moo 2, Phetkasem Road, Salui Sub-District, Thasae District, Chumporn 86140, Thailand; nirawat_me11@hotmail.com; 4 Clemson Veterinary Diagnostic Centre, Clemson University, 500 Clemson Road, Columbia, SC 29229, USA; wmanatc@clemson.edu

**Keywords:** oil palm agroecosystems, predator-based pest control, ecological infrastructure, sustainable agriculture

## Abstract

Barn owls (*Tyto alba*) are widely used as biological control agents in Southeast Asian agroecosystems, especially in oil palm plantations where rodent pests cause major yield losses. The success of such programs relies not only on nest box installation but also on maintaining the structural condition of these boxes. We analyzed monthly nest box monitoring data from January 2022 to May 2023 across five oil palm plantations (CPI1–CPI5) in Southern Thailand, including numbers of total, damaged, repaired, and unrepaired boxes. Substantial spatial variation was observed: CPI1 maintained the highest number of boxes (289) with a very low damage rate (~1.2%) and consistent repairs, whereas CPI4 showed the highest proportion of damaged boxes (~11%) and no repair activity. Chi-square and Kruskal–Wallis tests confirmed significant differences in damage rates among plantations (*p* < 0.001), although monthly variation was not statistically significant (*p* = 0.42). Visual inspection indicated increased deterioration during the wet season, suggesting weather-related stress on wooden structures. These results highlight the importance of maintaining nest box infrastructure as part of plantation management practices that support barn owl presence in oil palm agroecosystems. Keeping boxes functional throughout the year helps sustain a nature-based pest control service, reducing reliance on rodenticides and enhancing agroecosystem sustainability under humid tropical conditions.

## Simple Summary

This study examined the condition of barn owl nest boxes in five oil palm plantations in Southern Thailand between January 2022 and May 2023. Barn owls are commonly used to control rodent pests in oil palm systems, but the effectiveness of this approach depends on maintaining the nest boxes in good condition. The results showed clear differences among plantations. CPI1 had the largest number of nest boxes and the lowest damage rate, with repairs carried out regularly. In contrast, CPI4 had the highest proportion of damaged boxes and no evidence of repair activity. Statistical tests confirmed that damage rates differed significantly among plantations, although monthly variation was not significant. Field observations suggested that box deterioration increased during the rainy season, likely due to weather exposure affecting wooden structures. Overall, the findings highlight that regular maintenance of nest boxes is essential to support long-term predator-based rodent control and to reduce dependence on chemical rodenticides in tropical oil palm systems.

## 1. Introduction

Barn owls (*Tyto alba*) are among the most widely distributed avian predators, inhabiting ecosystems from grasslands and agricultural fields to urban areas across all continents except Antarctica [1,2]. Their ecological role as specialized rodent hunters is well established, supported by adaptations such as exceptional nocturnal vision, precise auditory localization, and silent flight that enable highly efficient predation [3]. As specialized rodent predators, barn owls are associated with the suppression of rodent activity in simplified agricultural landscapes where predator diversity is reduced and outbreaks are common [4]. In oil palm systems, their role has been framed as a biological control component within integrated pest management programs, potentially reducing the need for intensive rodenticide application [5,6].

Oil palm (*Elaeis guineensis*) plantations are among the most extensive monocultures in Southeast Asia, and rodent pests such as *Rattus tiomanicus* cause severe yield losses, estimated at 10–15% when uncontrolled [6,7]. Conventional control methods frequently involve anticoagulant rodenticides, including both first-generation and second-generation compounds. These substances can persist in the tissues of poisoned rodents and may cause secondary poisoning when predators or scavengers consume contaminated prey, potentially exposing non-target wildlife such as raptors to toxic residues [8,9,10]. The extent and type of rodenticide use may vary among plantations and was not directly quantified in this study.

Oil palm plantations in Southeast Asia increasingly function as tree–crop agroforestry systems, where managers integrate ecological components (e.g., barn owls, nest boxes, groundcover, riparian buffer strips) to stabilize production and reduce reliance on chemical control. In Malaysia, Indonesia, and Thailand, the installation of barn owl nest boxes has been widely promoted within oil palm plantations as part of integrated pest management programs aimed at reducing rodent damage. Because natural nesting cavities are scarce in monoculture landscapes, plantation managers commonly install artificial nest boxes to encourage barn owl colonization [11,12].

The success of barn owl programs in plantations depends on the provision of artificial nest boxes, as natural cavities are scarce in monoculture landscapes [13,14]. Numerous studies have shown that barn owls readily occupy artificial nest boxes and that their presence is associated with reductions in rodent activity [15]. Research has also addressed occupancy, diet composition, and reproductive ecology, including seasonal breeding dynamics [16]. However, an often overlooked but fundamental prerequisite for sustaining owl populations is the physical condition of nest boxes themselves.

Nest box condition—defined as the structural availability of boxes, including those damaged, repaired, or unrepaired—determines the nesting opportunities for owls. In tropical agroecosystems, high rainfall, humidity, and termite activity can accelerate deterioration of wooden boxes [17]. Accumulation of damaged boxes may, therefore, reduce the availability of suitable nesting structures within plantations. Although this study did not measure occupancy directly, structural deterioration may limit nesting opportunities if not addressed through maintenance. Moreover, differences in plantation-level management practices may lead to substantial variation in nest box conditions across estates, with important implications for the viability of barn owl populations and their role as biological control agents [18,19].

Despite widespread deployment of nest boxes across Southeast Asia, systematic assessments of their condition and maintenance remain scarce. Most prior work has focused on occupancy rates and breeding ecology, while overlooking the infrastructure required to sustain barn owl populations. To address this gap, the present study analyzes monthly nest box monitoring records from five oil palm plantations in Southern Thailand between 2022 and 2023. Specifically, we aim to (i) quantify the proportion of damaged, repaired, and unrepaired nest boxes; (ii) assess spatial variation among plantations with differing management practices; and (iii) evaluate seasonal tendencies in nest box deterioration. We hypothesized that nest box condition would differ significantly among plantations due to management practices, and that deterioration would increase during wet season months, reflecting weather-related stress on wooden structures. By examining nest box condition as a management variable, this study provides baseline information that may assist plantation managers in maintaining nest box infrastructure within oil palm agroecosystems.

## 2. Materials and Methods

### 2.1. Study Area

This study was conducted across five commercial oil palm (*Elaeis guineensis*) estates in Southern Thailand, representing managed tropical agroecosystems that integrate biological control components within crop production. Three sites (CPI1, CPI2, CPI3) were situated in Chumphon Province, while two sites (CPI4, CPI5) were in Prachuap Khiri Khan Province. The region is characterized by a tropical monsoon climate with a mean annual temperature of 27–28 °C and two distinct seasons: wet (May–October) and dry (November–April). Plantations differed in size and management practices. CPI1 was the largest estate (10,201 rai; ~1632 ha; 284–289 nest boxes installed), followed by CPI2 (3744 rai; ~599 ha; 60 boxes), CPI3 (1837 rai; ~294 ha; 51 boxes), CPI4 (2313 rai; ~370 ha; 20 boxes), and CPI5 (962 rai; ~154 ha; 32 boxes). Artificial nest boxes were installed independently by plantation managers as part of integrated pest management (IPM) programs to encourage colonization by barn owls and enhance biological control of rodents [5,6,13].

### 2.2. Nest Box Program and Definition of Condition

Nest boxes were constructed primarily of plywood and mounted on oil palm trunks or poles distributed across plantation blocks. The boxes had approximate dimensions of ~60 × 40 × 40 cm (height × width × depth), following designs commonly used for barn owl nest boxes in oil palm agroecosystems. Damaged boxes were defined as those showing structural deterioration such as cracks, partial collapse, water leakage, or termite infestation. These conditions were considered likely to compromise structural integrity and reduce the suitability of the boxes as nesting structures for barn owls. For the purpose of this study, nest box condition was defined as the structural and functional status of boxes, categorized into:

**Total nests**—number of boxes installed;
**Damaged**—boxes showing cracks, collapse, water leakage, or termite infestation, rendering them unsuitable for owl breeding [17];
**Repaired**—boxes repaired or replaced during the inspection month;
**Remaining**—damaged boxes not yet repaired after inspection.

### 2.3. Data Collection

Monitoring was conducted monthly between January 2022 and May 2023. The monitoring period corresponds to the available nest box inspection records maintained by plantation staff during routine monitoring programs. Plantation staff, trained under agricultural extension and conservation NGO programs, performed standardized nest box inspections. Protocols included visual checks of each box, photographic documentation, and classification into the four condition categories. No handling, capturing, or disturbance of owls or other wildlife occurred. Unlike many barn owl studies that include reproductive parameters (e.g., clutch size, fledging success) [16], this dataset focused exclusively on nest box condition.

### 2.4. Derived Variables and Data Processing

Because plantation size and the number of nest boxes differed among sites, damage and repair rates were analyzed as percentages relative to the total number of nest boxes within each plantation. To enable comparisons across sites and months, the following derived indicators were calculated:

% Damaged = (Damaged ÷ Total nests) × 100

% Repaired = (Repaired ÷ Total nests) × 100

Data were organized by plantation site (CPI1–CPI5) and month (January 2022–May 2023) to allow both spatial and temporal analyses.

### 2.5. Statistical Analyses

All analyses were performed in R version 4.4.1 (R Core Team, Vienna, Austria; https://www.R-project.org/). Data import and wrangling were conducted using the tidyverse version 2.0.0 and readxl version 1.4.5 packages, and descriptive statistics (means ± SD, maximum values, and temporal medians) were computed to summarize nest box condition. To test for spatial heterogeneity, a Chi-square test of independence was applied to proportions of damaged versus intact boxes, while non-parametric Kruskal–Wallis tests were used to assess differences in % damaged across plantations and months. Significant effects were followed by Dunn’s post hoc tests with Holm adjustment [14,15]. Seasonal tendencies were evaluated by pooling months into wet and dry seasons and applying a permutation test with 10,000 iterations to compare medians, ensuring robust estimation of *p*-values. Effect sizes were reported as epsilon-squared (ε^2^) for Kruskal–Wallis and rank-biserial correlation for pairwise comparisons, using the rstatix version 0.7.3 package. Temporal trends were visualized with line plots and seasonal differences with boxplots generated in ggplot2 version 4.0.1. All tests were two-tailed with significance set at *p* < 0.05.

## 3. Results

### 3.1. Overall Nest Box Condition

Across the five plantations (CPI1–CPI5), a total of 452 nest boxes were monitored monthly from January 2022 to May 2023. The proportion of damaged boxes varied substantially among sites. CPI1, the largest plantation, maintained the lowest average proportion of damaged boxes (1.2%), whereas CPI4 exhibited the highest proportion (11.2%) with no repairs conducted during the monitoring period. CPI2, CPI3, and CPI5 showed intermediate levels of damage (1.9–6.7%). Repair activities were concentrated in CPI1 and CPI2, while CPI3, CPI4, and CPI5 reported little to no repairs (Table 1).

### 3.2. Spatial Differences Among Plantations

A Chi-square test of independence revealed highly significant differences in the proportion of damaged boxes among plantations (χ^2^ = 201.3, df = 4, *p* < 0.001). Post hoc standardized residuals indicated that CPI4 contributed disproportionately to the overall association, with a significantly higher number of damaged boxes than expected, while CPI1 had significantly fewer.

**Figure 1:**
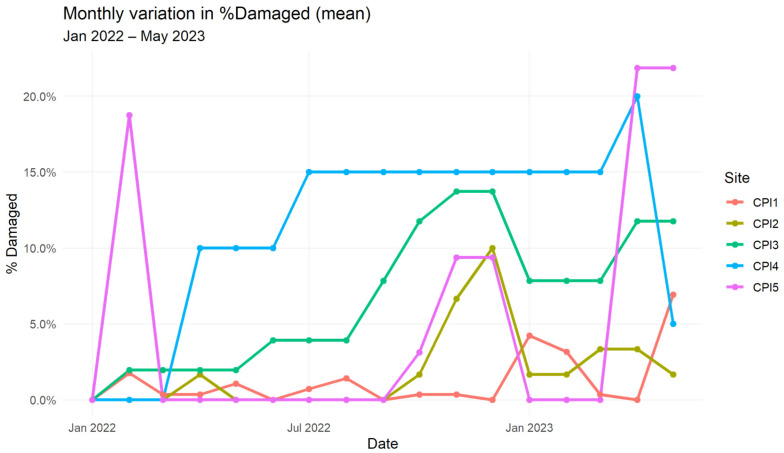
Monthly variation in percentage of damaged nest boxes across plantations (January 2022–May 2023). Lines indicate the mean percentage of damaged nest boxes per plantation site across the monitoring period.

Non-parametric testing confirmed spatial heterogeneity: Kruskal–Wallis analysis of % damaged across sites was significant (H = 27.5, df = 4, *p* < 0.001). Post hoc Dunn’s tests (Holm-adjusted) showed that CPI4 differed significantly from CPI1, CPI2, and CPI5 (*p* < 0.01) but not from CPI3 (Figure 1).

### 3.3. Temporal and Seasonal Trends

Monthly variation in % damaged was modest within each plantation. Across all sites combined, the Kruskal–Wallis test comparing % damaged across months was non-significant (H = 5.1, df = 4, *p* = 0.42). However, descriptive patterns suggested a tendency for higher damage during the wet season (May–October), particularly in CPI3 and CPI4, where proportions increased up to 10–15%.

**Figure 2:**
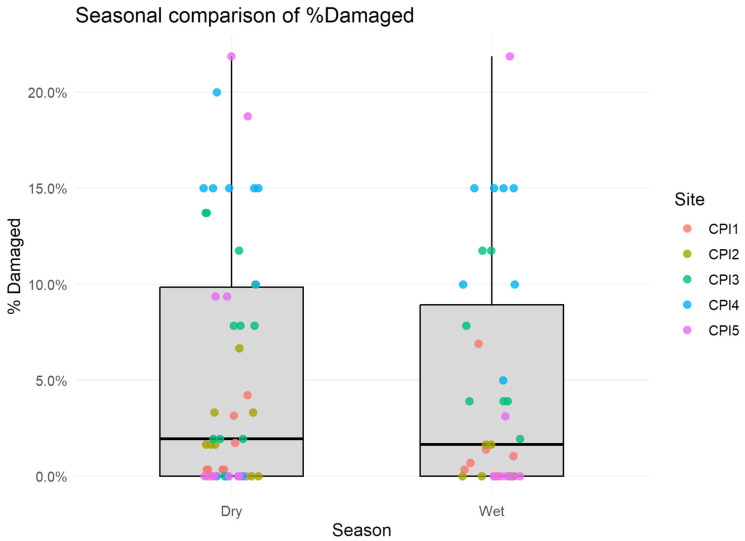
Comparison of % damaged between wet and dry season months across all plantations. Points represent plantation-level monthly medians; boxplots summarize overall distribution.

A permutation test comparing pooled wet versus dry season data supported a weak seasonal tendency, with higher % damaged in wet season months (median difference = +2.4%), though the difference did not reach statistical significance (*p* = 0.08; Figure 2).

### 3.4. Repair Dynamics

Repair activity was concentrated in CPI1 and CPI2, with very limited repairs in CPI3 and CPI5. CPI1 showed consistent repairs, averaging 0.9% per month, with repair events closely tracking monthly increases in damaged boxes. CPI2 recorded sporadic repairs (≤1% per month), while no repairs were documented in CPI3–CPI5 during the study period. These patterns suggest that variation among plantations may be related to differences in maintenance practices. However, management inputs were not quantified, and other factors such as plantation size, box age, or nest box density may also influence nest box condition. In the context of oil palm agroforestry, this means that the continuity of a nature-based rodent-control service is a management outcome and not merely an ecological accident.

## 4. Discussion

### 4.1. Management Practices Are the Primary Determinant of Nest Box Availability

Our results demonstrate pronounced differences in nest box condition across plantations. CPI1, with nearly 300 boxes and a regular repair program, maintained the lowest mean damage rates (1.2%), while CPI4, lacking maintenance, recorded the highest proportion of damaged boxes (11.0%). This pattern underscores that estate-level management practices, particularly inspection and repair, are decisive for maintaining functional nest box capacity. Similar findings have been reported in Malaysia, where nest box programs only sustained barn owl populations when combined with consistent upkeep [6,15]. Framed within the concept of “ecological infrastructure”, nest boxes form part of the structural infrastructure supporting predator presence, which depends on governance and resource allocation as much as it depends on biological processes [18,19].

### 4.2. Spatial Heterogeneity Reflects Plantation-Level Differences

Statistical analyses confirmed significant heterogeneity in damage rates among sites, with CPI4 disproportionately contributing to the overall differences. These results are consistent with previous reports showing that variation in adoption, density, and condition of nest boxes across estates translates into different levels of barn owl occupancy and breeding success [13,14]. Plantations that commit to structured nest box programs appear to buffer against deterioration, whereas those with minimal maintenance rapidly lose functional resources. Such variation highlights the need for standardized maintenance protocols across estates to ensure that barn owl programs deliver consistent pest management services.

### 4.3. Seasonal Stress Is Detectable but Secondary

Although overall monthly variation was modest and not statistically significant, descriptive patterns suggested greater deterioration during the wet season, especially in CPI3 and CPI4. This is consistent with prior work showing that rainfall, humidity, termite activity, and other wood-damaging insects such as ants can accelerate deterioration of wooden nest boxes [17]. However, because CPI1 maintained low damage rates year-round despite exposure to the same climatic regime, our findings suggest that management practices, rather than weather, represent the dominant driver of nest box condition. Seasonal weather, therefore, acts more as a stress multiplier: well-managed plantations can buffer climatic effects, while poorly managed plantations show amplified deterioration.

### 4.4. Implications for Integrated Pest Management (IPM)

Barn owls are widely recognized as effective biological control agents in oil palm landscapes, with diets dominated by rodent pests and demonstrating reductions in crop damage where populations are supported [4,5,14]. However, reliance on anticoagulant rodenticides remains widespread, despite well-documented risks of secondary poisoning and population declines in non-target wildlife [8,9,10].

Although barn owl-based biological control has been widely promoted in oil palm agroecosystems, maintaining nest box infrastructure requires periodic inspection, repair, and labor inputs. These operational costs must typically be borne by plantation managers. In comparison, rodenticides may sometimes be perceived as a faster or more predictable control method. Consequently, many plantations continue to use chemical rodenticides alongside biological control rather than replacing them entirely. Recognizing these economic and operational considerations is important when evaluating the adoption and long-term sustainability of predator-based pest control strategies.

Our findings suggest that ensuring functional nest boxes through regular inspection and timely repairs may support biological control programs. However, because rodenticide use was not quantified in this study, the relationship between nest box maintenance and reduced chemical control remains uncertain. Investment in maintenance may help maintain the availability of nesting structures for barn owls within plantation landscapes.

Despite documented associations between barn owl presence and reduced rodent activity in some plantation systems, chemical rodent control remains widely practiced. This may reflect several non-ecological factors, including perceived immediacy of rodenticide effects, labor constraints associated with nest box inspection and repair, risk aversion among managers, and institutional inertia within established pest control protocols. Thus, continued rodenticide use does not necessarily contradict the potential contribution of barn owls but may indicate that biological control is implemented as a complementary rather than exclusive strategy.

### 4.5. Ecosystem Service and Environmental Sustainability Implications

Maintaining nest box infrastructure helps preserve the structural conditions necessary for predator-based pest control programs [11,12], which may contribute to reducing reliance on rodenticides and associated environmental contamination. In tropical agroecosystems, where chemical control remains widespread, this maintenance acts as an indirect pollution-prevention measure and contributes to sustainable intensification goals. Quantifying the environmental and economic co-benefits of such management interventions represents a valuable next step for integrating biodiversity-based practices into agricultural policy frameworks.

### 4.6. Operational Guidelines for Ecosystem Service Maintenance

Translating these findings into practice, we propose operational guidelines for maintaining ecological infrastructure that underpins predator-based pest control services in oil palm agroecosystems. This should include (i) design improvements to reduce exposure to rainfall and slow structural deterioration, including damage caused by termites [17], (ii) density and placement alignment with ecological guidelines [5,18], and (iii) scheduled inspections—minimally before and after the wet season—with repairs completed within 30 days. Such a standard would align with current calls to view barn owl nest boxes as ecological infrastructure, requiring continuous investment rather than one-off installation [18,19].

### 4.7. Implications for Agroforestry Design and Management

For agroforestry planners, our results indicate that barn owl nest boxes should be treated similarly to other long-lived structural components (e.g., shade trees, live fences, perches) whose functionality decays under tropical weathering. A two-season inspection schedule (pre- and post-wet season) would likely improve functional retention of boxes, thereby safeguarding predator presence and potentially reducing the need for rodenticide use, although empirical validation across multiple years would be required to quantify its effectiveness. Embedding such a schedule into routine plantation operations converts a single-species wildlife measure into a stable ecosystem service component of the wider agroforestry system.

### 4.8. Limitations and Research Needs

Although the dataset spans a single annual cycle, it captures the full wet–dry seasonal variation characteristic of humid tropical systems. This provides a representative baseline for evaluating management-driven patterns of nest box deterioration and repair. Extending the monitoring to multiple years would refine temporal estimates but is unlikely to change the central inference that management practices govern service continuity. Moreover, data were confined to nest box condition; we did not measure occupancy, reproduction, or rodent damage reduction directly. Because owl occupancy, predation rates, rodent damage, and rodenticide use were not quantified in this study, the ecological outcomes of nest box maintenance should be interpreted cautiously. Future studies integrating nest box condition with owl occupancy, predation rates, and rodent damage assessments would help clarify the ecological consequences of nest box maintenance programs. Monitoring rodenticide use and residues in pellets would also help clarify risks to barn owl populations [9,10]. Establishing a standardized reporting framework—including % damaged, % repaired, functional availability, and links to occupancy and pest suppression—would facilitate benchmarking across estates and accelerate learning regionally.

### 4.9. Economic and Environmental Co-Benefits

Although this study did not quantify rodenticide use or cost savings directly, maintaining functional nest boxes may support plantation-level strategies that aim to integrate biological and chemical control methods. Where barn owl programs are implemented, structural maintenance of nest boxes helps preserve the ecological infrastructure required for predator presence. Future research should explicitly compare the financial costs of nest box inspection and repair with expenditures on rodenticide purchase and application as well as evaluate potential environmental trade-offs associated with chemical control [9,10]. Such analyses would clarify whether sustained infrastructure maintenance offers measurable economic and ecological advantages under tropical plantation conditions.

## 5. Conclusions

This study provides a systematic assessment of barn owl nest box condition in oil palm plantations in Southern Thailand. Monitoring of 452 boxes across five estates during 2022–2023 revealed marked spatial heterogeneity: CPI1, with a structured repair program, maintained consistently low damage rates, whereas CPI4, lacking maintenance, recorded the highest proportion of damaged boxes. Although descriptive patterns suggested greater deterioration during the wet season, management practice—particularly the presence or absence of regular inspections and repairs—emerged as the dominant determinant of functional nest box availability.

These findings underscore that barn owl-based integrated pest management requires more than nest box installation. Sustained maintenance regimes, including inspections and timely repairs, are essential to ensuring the continued availability of functional nesting structures that may support barn owl presence in plantation landscapes. For managers of tree–crop agroforestry systems, this implies that modest, recurrent investments in monitoring and repairs yield greater ecological and economic returns than simply expanding nest box numbers, because they help maintain the availability of suitable nesting structures for barn owls within oil palm production systems. For researchers, the next step is to integrate nest box condition monitoring with occupancy, reproduction, rodent damage assessments, and trials of alternative materials and designs. Embedding maintenance standards into plantation operations will enhance the reliability and scalability of barn owl programs as a sustainable, nature-based solution for rodent control in tropical agroecosystems. Although derived from a single-year dataset, these findings establish a replicable framework for linking ecological infrastructure maintenance with ecosystem service delivery in tropical agroecosystems, providing a basis for future monitoring and research on nest box management in oil palm agroecosystems.

## Figures and Tables

**Table 1:** Summary of nest box condition across five oil palm plantations in Southern Thailand (January 2022–May 2023).

Plantation	Total Nests	Mean % Damaged ± SD	Max % Damaged	Mean % Repaired ± SD	Notes
CPI1	289	1.2 ± 1.9	6.9	0.7 ± 1.2	Regular repair schedule
CPI2	60	1.9 ± 2.8	10	0.4 ± 0.9	Occasional repairs
CPI3	51	6.7 ± 4.6	13.7	0.3 ± 1.4	Occasional repairs
CPI4	20	11.2 ± 6.3	20	0	No repairs
CPI5	32	5.0 ± 8.2	21.9	1.1 ± 4.5	Occasional repairs

## Data Availability

Data is contained within the article or Supplementary Materials.

## Supplementary Materials

The following supporting information can be downloaded at https://www.mdpi.com/article/10.3390/ani16060881/s1, R script used for data analysis and generation of Figure 1 and Figure 2.
